# Metabolic signatures of immune cells in chronic kidney disease

**DOI:** 10.1017/erm.2022.35

**Published:** 2022-10-21

**Authors:** Jie Li, Yi Yang, Yanan Wang, Qing Li, Fan He

**Affiliations:** Department of Nephrology, Tongji Hospital Affiliated to Tongji Medical College, Huazhong University of Science and Technology, Wuhan 430030, China

**Keywords:** Chronic kidney disease, fibrosis, immune cells, immunometabolism, metabolism

## Abstract

Immune cells play a key role in maintaining renal dynamic balance and dealing with renal injury. The physiological and pathological functions of immune cells are intricately connected to their metabolic characteristics. However, immunometabolism in chronic kidney disease (CKD) is not fully understood. Pathophysiologically, disruption of kidney immune cells homeostasis causes inflammation and tissue damage via triggering metabolic reprogramming. The diverse metabolic characteristics of immune cells at different stages of CKD are strongly associated with their different pathological effect. In this work, we reviewed the metabolic characteristics of immune cells (macrophages, natural killer cells, T cells, natural killer T cells and B cells) and several non-immune cells, as well as potential treatments targeting immunometabolism in CKD. We attempt to elaborate on the metabolic signatures of immune cells and their intimate correlation with non-immune cells in CKD.

## Introduction

Chronic kidney disease (CKD) is tightly associated with high levels of morbidity and mortality, the global prevalence of CKD is estimated at about 14% (Ref. 1), which poses a significant threat to human health and economic burden especially when it progresses to uraemia (Ref. 2). The aetiology of CKD includes autoimmune disease, hypertension, diabetes mellitus, infections, polycystic kidney disease and hereditary diseases (Refs 3, 4). It is worth mentioning that the immune system plays a crucial role in most forms of kidney injury, such as in diverse glomerular nephritis (GN) and IgA nephropathy (IgAN) (Ref. 5). In immune-mediated kidney diseases, immune cells are activated and respond to the stimuli. Sustained immune response based on inflammatory mediators including complement, reactive oxygen species and cytokines leads to persistent renal structural and functional damage, and ultimately causes irreversible renal tubulointerstitium fibrosis (Refs 6, 7, 8).

**Fig. 1.**
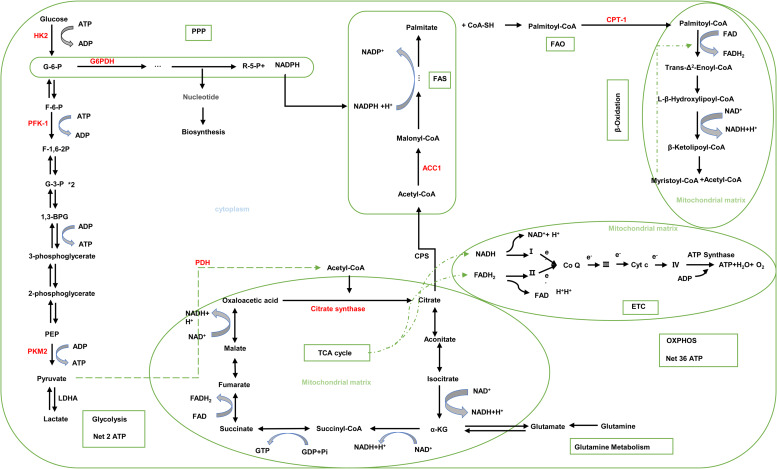
Six main metabolic pathways in cells. Glucose is used to produce ATP by glycolysis and OXPHOS. G-6-P is catabolised to R-5-P and NADPH through the PPP. TCA cycle, ETC and *β*-oxidation occur in the mitochondrial matrix, while glycolysis, FAO, FAS and glutamine metabolism occur in the cytoplasm. The intermediates of the TCA cycle are interconnected with glutamine metabolism, glycolysis and FAS. Abbreviations: R-5-P, ribose 5-phosphate; PPP, pentose phosphate pathway; FAS, fatty acid synthesis; FAO, fatty acid oxidation; G6PDH, glucose-6-phosphate dehydrogenase; HK2, hexokinase 2; PFK-1, phosphofructokinase 1; F-6-P, fructose 6 phosphate; F-1,6-2P, fructose 1,6 diphosphate; G-3-P, glyceraldehyde triphosphate; G-1,3-2P, 1,3-bisphosphoglycerate; PKM2, pyruvate kinase isozymes M2; PDH, pyruvate dehydrogenase; PEP, phosphoenolpyruvate; LADH, lactate dehydrogenase A; ACC1, acetyl-CoA carboxylase 1; CPT-1, carnitine palmitoyl transferase 1; CPS, citrate-pyruvate shuttle; *α*-KG, *α*-ketoglutarate; ETC, electron transport chain; I, II, III, IV; respiratory chain enzyme complexes I, II, III, IV; Cyt c, cytochrome c; Co Q, coenzyme Q. Red letters indicate rate-limiting enzyme.

The kidney is frequently the target organ of autoimmune and systemic immune disorders. Immune responses mediated by resident immune cells or circulating recruited immune cells lead to renal damage and clinical symptoms. In kidney diseases, immune cells such as T cells, macrophages and dendritic cells (DCs) are persistently activated during the whole process. Importantly, increasing evidence indicates that the proliferation and activation of immune cells are regulated by the metabolic pathways (Ref. 9). Under the condition of homeostasis, the six main metabolic pathways including glucose, fatty acid and amino acid metabolism cooperate with each other to maintain the stability of immunometabolism (Fig. 1) (Refs 9, 10). However, pathophysiologically, metabolic reprogramming of immune cells was triggered.

Here we mainly introduce the immune response-mediated CKD and explore the changes in the metabolism of immune cells and non-immune cells during the progression of the disease, and expect to provide a novel direction for the remedy and retard the progression.

## Immune cells and non-immune cells in CKD

The kidney immune cells include resident immune cells such as macrophages and DCs, and circulating recruited immune cells such as neutrophils, natural killer (NK) lymphocytes, natural killer T (NKT) lymphocytes, T lymphocytes and B lymphocytes (Refs 5, 10). The function of kidney immune cells has been well reviewed (Refs 11, 12, 13, 14, 15, 16). Kidney parenchymal cells include endothelial cells, tubular epithelial cells (TECs), mesangial cells and podocytes (Ref. 5). Those parenchymal cells intimately cooperate with immune cells to maintain renal homeostatic condition (Refs 2, 3). Under pathological conditions, kidney parenchymal cells express a subset of Toll-like receptors and induce an innate immune response. Immune cells mediate renal inflammation by presenting antigens (macrophages, DCs) to T cells, producing cytokines (tumour necrosis factor (TNF)-*α*, interleukin, interferon (IFN)-*γ*), chemokines, inducible nitric oxide synthase, hyaluronic acid, and so on (Ref. 17). Eventually, kidney damage and immune-related kidney diseases can occur.

## Immune cells and non-immune cells and their metabolic characteristics in CKD

### The role of dendritic cells and macrophages in CKD and their metabolic characteristics

**Fig. 2.**
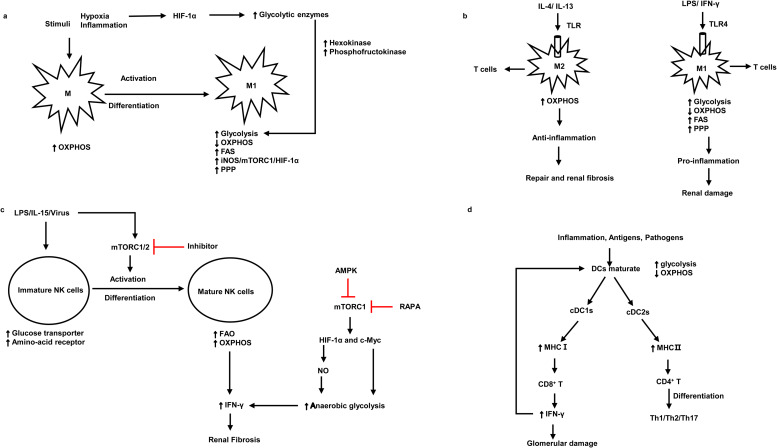
The association between the pathogenic roles and metabolic signatures of macrophages, NK cells and DCs. (a) The metabolism signatures of macrophages and the metabolism signatures when they activate and differentiate into M1. (b) Metabolism characteristics of M1 and M2 under different antigen stimulation and corresponding kidney damage. (c) The activation and differentiation of immature NK cells and metabolism characteristics of immature NK cells and mature NK cells. (d) The metabolism characteristics of mature DCs and the interaction between DCs and T cells. M1, macrophage 1; M2, macrophage 2; mTORC1, mammalian target of rapamycin complex 1; HIF-1*α*, hypoxia-inducible factor 1*α*; OXPHOS, oxidative phosphorylation; FAS, fatty acid synthesis; PPP, pentose phosphate pathway; TLR, Toll-like receptor 4; LPS, lipopolysaccharides; NO, nitric oxide; cDC, conventional dendritic cells; NK cells, natural killer cells; iNOS, inducible nitric oxide synthase; AMPK, AMP-activated serine/threonine protein kinase; RAPA, rapamycin; MHC, major histocompatibility complex. Red lines indicate suppression.

Kidney resident antigen-presenting cells, macrophages and DCs are mostly enriched in the tubulointerstitium rather than the glomerulus (Refs 3, 18, 19, 20, 21). Numerous studies have demonstrated that DCs are pathogenic in the mouse model of adriamycin glomerulopathy and IgAN, indicating that DCs play an important role in the progression of kidney diseases (Refs 22, 23, 24, 25). CD103 + DCs enhance the activity of CD8 + T cells and promote glomerular injury (Refs 22, 26). Inhibition of CD103 + DCs attenuates kidney injury and fibrosis by reducing T helper 17 (Th17) cells and increasing regulatory T cells (Tregs) (Fig. 2d) (Refs 27, 28). As for macrophages, they are activated into M1 macrophages or M2 macrophages under different stimuli (Table 1) (Ref. 29). Most forms of renal inflammation are characterised by M1 macrophage infiltration in the early phase but M2 macrophage infiltration in the chronic phase. The persistence of M2 macrophages is strongly associated with renal fibrosis and progressive CKD (Refs 30, 31, 32, 33).

**Table 1.** The metabolic characteristics of macrophages

Macrophages subset	Induction	Metabolic programmes	Functions	Ref.
M1	LPS/IFN-*γ*/NF-*κ*B/MMIF/PAMP/DAMP/CRP/IL-1	↑ Glycolysis ↑ Fatty acid synthesis ↓ OXPHOS ↑ iNOS/mTORC1/HIF-1*α* ↑ PPP	Pro-immunology Renal damage	(Refs 3, 12)
M2	IL-4, IL-13/IL-10	↓Glycolysis ↑ Fatty acid oxidation ↑ OXPHOS ↑ Arginase/mTORC2 ↓ PPP	Anti-inflammatory Repair process Renal fibrosis	(Refs 3, 29, 30)

M1, macrophage 1; M2, macrophage 2; OXPHOS, oxidative phosphorylation; iNOS, inducible nitric oxide synthase; mTORC1, mammalian target of rapamycin complex 1; HIF-1*α*, hypoxia-inducible factor 1 *α*; PPP, pentose phosphate pathway; mTORC2, mammalian target of rapamycin complex 2; LPS, lipopolysaccharides; IFN-*γ*, interferon-*γ*; IL, interleukins; MMIF, macrophage migration inhibitory factor; PAMP, pathogen-associated molecular patterns; DAMP, danger-associated molecular patterns; CRP, C-reactive protein.

Under the stimulation of pro-inflammatory signals, M1 macrophages (Table 1) and DCs are activated and undergo a metabolic switch from oxidative phosphorylation (OXPHOS) to glycolysis (Table 2) (Refs 3, 18). It is thought that cells rely on pyruvate to convert to lactate only when oxygen is insufficient. However, growing evidence suggests that tumour cells use glycolysis rather than OXPHOS pathways for energy generation even in the presence of adequate oxygen availability for mitochondrial OXPHOS (Refs 34, 35). Likewise, stimulated immune cells use glycolysis under oxygen-rich conditions. Increased aerobic glycolysis is the key metabolic phenotype of polycystic kidney disease and renal cell carcinoma (Refs 36, 37). Oxygen levels and changes in nutrient availability influence the metabolic reprogramming of cells. Hypoxia-inducible factor 1*α* (HIF-1*α*) is crucial for this course, which products sustained adenosine triphosphate (ATP) by inducing the expression of glucose transporter and glycolytic enzymes (Refs 38, 39) (Fig. 2a). The phenotype switch of macrophages towards M1, there is an increase in glycolysis but a decrease in OXPHOS, which leads to renal injury in the early stage of kidney diseases. In contrast, M2 macrophages keep high OXPHOS and play a significant role in renal fibrosis in the chronic stage of kidney injury (Refs 40, 41) (Fig. 2b). Likewise, a recent study suggested that in lupus nephritis, macrophages metabolism in human and mouse undergoes a shift to glycolysis in answer to IgG immune complex-induced inflammation, this metabolic reprogramming was dependent on the mammalian target of rapamycin (mTOR) and HIF-1*α*, inhibition of glycolysis caused a decrease in the number of renal macrophages (Ref. 42). Another research found that *β*-activated kinase 1-binding protein 1, a transforming growth factor, can regulate glycolysis and activate macrophages via activating nuclear factor *κ*B (NF-*κ*B)/HIF-1*α* signalling pathway in diabetic nephropathy (Ref. 43). Besides, treatment with glycolysis inhibitors in the mouse model of unilateral ureteric obstruction (UUO) significantly reduces macrophage infiltration and renal interstitial fibrosis (Ref. 44), suggesting that regulating macrophage glycolytic metabolism is crucial for kidney disease progression.

**Table 2.** The metabolic characteristics of DCs and NK cells

DCs subset/NK cells	Activator	Induction	Metabolic characteristics	Ref.
Immature DCs	–	Cytokines PAMPs TCR NLR MHC class I/II	↑ Glycogen ↓ Glycolysis	(Ref. 3)
Mature DCs	TBK, NF-*κ*B, AKT, mTORC1, HIF-1*α*	–	↑ Glycolysis	(Ref. 3)
Immature NK cells	–	–	↑ Glucose transporter ↑ Nutrient receptor	(Ref. 50)
Mature NK cells	Viruses or cytokines	–	↑ FAO ↑Aerobic metabolism ↓ Glycolysis ↓cellular glucose uptake	(Ref. 50)

DCs, dendritic cells; NK cells, natural killer cells; PAMPs, pathogen-associated molecular patterns; TCR, T cell receptor; NLR, NOD-like receptor; MHC, major histocompatibility complex; TBK, serine/threonine-protein kinase; NF-*κ*B, nuclear factor *κ*B; AKT, RAC-*α* serine/threonine protein kinase; mTORC1, mammalian target of rapamycin complex 1; HIF-1*α*, hypoxia-inducible factor 1*α*; FAO, fatty acid oxidation.

### NK cell and its metabolism in CKD

NK cells are one of the specialised subpopulations of innate lymphoid cells (Ref. 45). They play a key role in the immune response to anti-virus, killing tumours, aseptic inflammation, as well as kidney allograft rejection, and produce inflammatory cytokines (TNF-*α*, IFN-*γ*) and chemokines (MIP-1*α*, MIP-1*β* and RANTES) (Ref. 46). Some evidence suggested that NK cells participate in acute kidney ischaemia injury and kidney allograft rejection (Refs 13, 47, 48). Recently, Law *et al*. demonstrated that human tubulointerstitial CD56^bright^ NK cells lead to the progression of CKD and kidney fibrosis by the production of proinflammatory cytokine IFN-*γ* (Ref. 49). IFN-*γ* can also induce proinflammation in kidney parenchymal cells and promotes M1 activation to exert pro-inflammation responses (Ref. 13).

During the activation and differentiation of NK cells, metabolism changed. Immature NK cells upregulate glucose transporter and nutrient receptor expression. Whilst upon the use of interleukin-15 (IL-15) in vitro, immature NK cells differentiate and activate to be mature NK cells, which increases fatty acid oxidation (FAO) and OXPHOS (Table 2) (Refs 50, 51). Upregulated OXPHOS pathway is critical for the production of IFN-*γ* for activation-induced NK cells. Furthermore, the metabolic regulator mTOR is critical for IL-15 signalling during the development and activation of NK cells and the production of IFN-*γ*. mTOR deficiency deeply impaired the activation of NK cells in the early stage (Ref. 50). mTOR is known to integrate cellular growth and metabolism via boosting cell proliferation and glycolysis (Ref. 52). mTORC1 is a complex composed of mTOR and other three subunits, which participate in glycolysis by facilitating the expression of HIF-1*α* and c-Myc. Its inhibitor rapamycin abrogates inflammation-induced priming of NK cells (Ref. 50). The energy sensor AMP-activated serine/threonine protein kinase (AMPK) also inhibits mTORC1 and thus inhibits the production of INF-*γ* (Ref. 52), therefore alleviating renal fibrosis (Ref. 49) (Fig. 2c).

### T cells and their metabolism in CKD

T lymphocytes, the major effector cells in cellular immunity, produce cytokines in responses to mediate inflammation and coordinate other types of immune cells. In kidney diseases, T cells are well known for causing acute and CKDs, particularly in immune-mediated renal disease. Multiple evidence had shown that T cells play crucial roles in the initiation and progression of kidney diseases (Refs 53, 54, 55).

Recently, the correlation between the cellular metabolism of T cells and their function has been increasingly emphasised. In quiescent cells such as naïve T and memory T (Tm) cells, catabolic metabolism of glucose and amino acid facilitates its energy and biosynthesis requirement (Refs 56, 57). After activation, T cells undergo drastic changes in function, rapid cellular growth, and the burst of cellular proliferation are engaged in (Ref. 58). T cells reprogramme their metabolic pathways from mitochondrial OXPHOS, fatty acids *β*-oxidation to aerobic glycolysis, pentose-phosphate and glutaminolysis pathways. Activated T cells go from producing large amounts of ATP to producing sufficient ATP and a mass of intermediates for the generation of biomass (Ref. 59). The mTOR plays a key role in glycolytic metabolism (Refs 58, 60). mTOR complex 1 (mTORC1) participates in the induction of T cells glycolysis and holds aerobic glycolysis of effector T cells (Refs 59, 60, 61). A mouse experiment found that treatment with rapamycin, an allosteric inhibitor of the mTOR, not only reduces the proportion of Th1, Th2 and Th17 cells but also boosts DCs to alleviate kidney damage in BALB/C mice with systemic lupus erythematosus (SLE) (Ref. 62). Furthermore, HIF-1*α* is critical for the activation of T cells (Ref. 63). Under the stimuli of various cytokines and molecules, a certain T cell subset was activated, and the metabolism changed (Table 3). Under the induction of IL-12 or T-bet, naïve T cells differentiate into Th1 cells, which perform a high OXOHOS and glycolysis metabolism and prevent renal fibrosis (Refs 56, 64). Activation of the Th2 and T follicular helper cells also predominately utilises OXOHOS and glycolysis metabolism to promote renal fibrosis under diverse stimuli (Refs 56, 64, 65, 66). Activated Th17 cells exploit glycolysis, OXOHOS and fatty acid synthesis (FAS) to exert pro-fibrosis function (Refs 56, 64, 65). Tm and naïve cells make full use of OXPHOS and FAO to sustain longevity (Refs 3, 56, 65). Besides, it has been proposed that suppression of acetyl-CoA carboxylase 1 restrains the FAS, thus inhibiting the glycolytic-lipogenic pathway, which facilitates Tregs development and curbs Th17 cell differentiation (Ref. 67).

**Table 3.** T cells subset and their metabolic characteristics

T cells subset	Induction	Metabolic characteristics	Secreted cytokines	Functions	Functions in kidney	Refs.
Th1	IL-12, T-bet	↑ Glycolysis ↑ OXPHOS	INF-*γ*, IL-1	Cellular immunity	Antifibrotic	(Refs 56, 64)
Th2	IL-25, IL-33, GATA-3	↑ Glycolysis ↑ OXPHOS	IL-4, IL-5, IL-13, IL-10	Humoral immunity, parasitic immunity, allergic disease	Renal fibrosis	(Refs 56, 64, 65, 66)
Th17	TGF-*β*/IL-6, IL-21/IL-23, IL-1, IL2(-), ROR*γ*t/ROR*α*, STAT3, SMADs, IRF4, BATF, c-Maf, RUNX1	↑ Glycolysis ↑ OXPHOS ↑ FAS	TGF-*β*, IL-6, IL-1*β*, IL-17, IL-21, IL-22, IL-23	Tissue inflammation, mucosal immunity, extracellular bacteria and fungi infection, autoimmunity, cancer	Renal fibrosis	(Refs 56, 64, 65)
Tfh	IL-6, IL-21, Bcl6, STAT3/BATF/ IRF4, Tox2, Blimp-1	↑ Glycolysis ↑ OXPHOS	IL-4, IL-21, CD40L, CXCR5	Germinal centre, immune-mediated disorders (infection, autoimmunity)	Renal fibrosis	(Refs 56, 64)
Treg cells	–	↑ OXPHOS ↑ Glycolysis ↑ FAO	CXCR5, IL-10, TGF-*β*	Tumour microenvironment, suppression of effector T cells, inhibit innate immune response, maintenance long-lived	IRI protection	(Refs 56, 64, 65, 66)
Tm cell	–	↑ OXPHOS ↑ FAO	–	Immune memory, longevity	–	(Refs 3, 56, 65)
Resting T cells	–	↑ OXPHOS	–	Longevity	–	(Ref. 3)
Tn cells	–	↑ OXPHOS ↑ FAO	–	Growth, longevity	–	(Ref. 56)

Th, T helper; Tfh, T follicular helper; Treg cells, regulatory T cells; Tn, naive T cell; TNF, tumour necrosis factor; Tm cell, memory T cells; IFN-*γ*, Interferon-*γ*; IL, iinterleukins; OXPHOS, oxidative phosphorylation; FAO, fatty acid oxidation; FAS, fatty acid synthesis; ROR, RAR-related orphan receptor; GATA-3, transcription factors GATA binding protein-3; Bcl6, B-cell CLL/lymphoma 6; TGF, tumour necrosis growth factor; STAT, selective transcription factor; IRF4, interferon regulatory factor 4; BATF, basic leucine transcription factor; c-Maf, transcription factor c-Maf; RUNX1, Runt-related transcription factor 1; SMAD, interacting transcription factor; IRI, ischaemia-reperfusion injury; Tox2, transcription factor Tox2; Blimp-1, transcription factor Blimp-1; CD40L, recombinant cluster of differentiation 40 ligand; CXCR, chemokine receptor.

### NKT cells and their metabolism in CKD

NKT cells are CD1d-restricted, glycolipid antigens-reactive T cells, which express semi-invariant T cell antigen receptor (TCR) and support cell-mediated immunity against infection, autoimmunity, cancer, allergy, allograft rejection and graft-versus-host disease (Refs 68, 69). NKT cells are divided into invariant natural killer (iNKT) cells and type II NKT cells two populations by surface markers and their receptor TCR usage (Ref. 14). In kidney ischaemia-reperfusion injury (IRI), iNKT cells and type II NKT cells play opposite roles. Inhibiting the production of IFN-*γ* by iNKT cells protects the kidney from IRI in murine model (Ref. 70). However, type II NKT cells protect the kidney by decreasing IFN-*γ* and IL-6 levels and enhancing IL-4 and IL-10 (Ref. 71). In the murine model of experimental crescent glomerulonephritis and anti-glomerular basement membrane (anti-GBM) glomerulonephritis, a study found a reduction of iNKT cells aggravates disease progression, whereas activation of iNKT ameliorates GN (Refs 72, 73, 74). Similarly, diminished iNKT cell counts are observed in patients with CKD, especially in end-stage renal disease (ESRD), which revert to normal levels after renal transplantation (Refs 75, 76). In an experimental model of lupus nephritis, repeated administration of *α*-GalCer to BWF1 mice ameliorates lupus disease activity and slows the development of lupus nephritis by suppressing Th2 immune responses in NKT cells (Ref. 77). However, Zeng *et al*. found administration of *α*-GalCer in BWF1 mice exacerbates lupus nephritis via inducing Th1-type immune responses (Ref. 78). These contradictory results were believed that influenced by the dose, dosing interval or the age of the mouse used, yet the molecular mechanisms behind these differences are not clear. Recently, NKT cells are reported to promote M2 macrophage-to-myofibroblast transition in kidney fibrosis by substantive IL-4 expression (Ref. 79).

Metabolic dysregulation changes the fate and function of iNKT cells, which is tightly associated with the progression of immune-related renal disease. Normally, activated effector T cells undergo a metabolic switch from OXPHOS to glycolysis and glutaminolysis to generate high levels of ATP for rapid proliferation and fuelling substrate biogenesis and effector functions (Refs 80, 81). Tm cells and Tregs utilise FAO metabolism for their survivorship and durability in nutrient-poor environments (Refs 82, 83), upon reactivation, Tm cells also switch to glycolytic metabolism and under polarisation to effector cells (Ref. 84). Nevertheless, metabolic pathways related to iNKT cells in immune-related diseases remain little known. Recently, a study demonstrated that after TCR stimulation, iNKT cells activate glycolysis, which in turn increases iNKT cell proliferation and IFN-*γ* production by promoting TCR recycling. Inhibiting glycolysis reduced the activity of protein kinase C and phosphoinositide 3-kinase–protein kinase B, which suppressed TCR accumulation, thus reducing the iNKT cells and immune injury (Ref. 85). Similarly, Webb *et al*. revealed that pretreatment with 2-deoxyglucose, cobalt chloride and AICAR, which decrease glycolysis, upregulate HIF-1*α* and activate AMPK, respectively, significantly enhanced iNKT cell activation (Ref. 86). Furthermore, Khurana *et al*. recently demonstrated that human iNKT cells utilise fatty acids but not glucose or glutamine as oxidative substrates exerting effector functions more than conventional T cells in the tumour microenvironment (Ref. 87). They possess a ‘memory-like’ metabolic phenotype but sustained high-level fatty acid metabolism upon stimulation. Nevertheless, how the metabolic programming alters the iNKT cells in CKD was not well elaborated.

### B cells and their metabolism in CKD

B lymphocytes consist of several subsets, including B1 cells, B2 cells, Bregs and memory B cells. B cells play a central role in renal autoimmune diseases and renal transplantation by producing autoantibodies such as anti-neutrophil cytoplasmic antibodies (ANCA), antinuclear antibody, anti-GBM antibody and IgA (Refs 88, 89, 90), presenting antigens to T cells (Ref. 91), and producing cytokines IL-10, transforming growth factor *β* (TGF-*β*) and IL-35 (Ref. 92). Regulatory B cells (Bregs) attenuate inflammation and conduce to the maintenance of immune tolerance. Bregs deficiency spoils renal function in the SLE, ANCA antibody-associated vasculitis, as well as in the renal transplant rejection and tolerance (Ref. 15).

The link between immunometabolism and autoimmunity has been extensively explored over the past decade. B cells, however, have received relatively little attention. Naïve B cells rely on FAO to generate ATP. During activation, OXPHOS, tricarboxylic acid (TCA) cycle and nucleotide biosynthesis increase, and FAO is downregulated (Refs 93, 94). Plasma cells, the immunoglobulins antibody-secreting cells, take up glucose via the hexosamine pathway. They also rely on long-chain fatty acids and glutamine as substrates for oxidative metabolism to feed basal OXPHOS (Refs 93, 95). However, few studies have directly explored the correlation between metabolism reprogramming and B cell-related kidney diseases. More work is needed to investigate the characteristics and consequences of metabolic dysregulation in autoimmune B cell-related kidney diseases.

## Metabolic characteristics in the progression of renal fibrosis

**Fig. 3.**
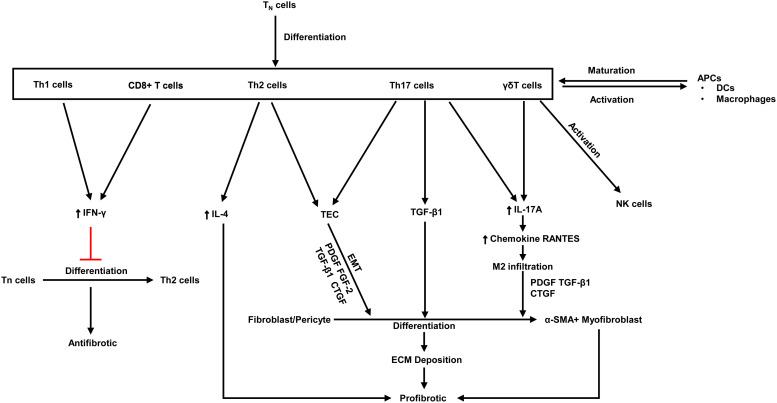
The antifibrotic and profibrotic functions of various T cell subsets. PDGF, platelet-derived growth factor; Th, T helper; ECM, extracellular matrix; FGF, fibroblast growth factor; CTGF, connective tissue growth factor; TGF, transforming growth factor; *α*-SMA, *α*-smooth muscle actin; IFN-*γ*, Interferon-*γ*; IL, Interleukins; Tn, naive T cell; APCs, antigen-presenting cells; DCs, dendritic cells; EMT, epithelial-mesenchymal transition. Red lines indicate suppression.

Fibrosis is characterised by aberrant extracellular matrix deposition, which leads to morbidity, dysfunction and even death. Upon activation of certain immune cells such as Th1 and CD8 + T cells restrains fibroblast-induced collagen synthesis and exerts an antifibrotic effect (Refs 96, 97). On the contrary, activation of Th2 cell, Th17 cell and *γδ* T cells plays a key role in kidney fibrosis (Ref. 96) (Fig. 3).

Nowadays, preventing kidney fibrosis by regulating cellular metabolism brings novel therapeutic opportunities. Recently, a study demonstrates that orphan nuclear receptor COUP-TFII, which is a key regulator of glucose and lipid metabolism, enhances myofibroblast glycolysis leading to kidney fibrosis (Ref. 98). Moreover, it is revealed that tuberous sclerosis complex 1, a negative regulatory factor of the mTORC1, promotes the progression of kidney interstitial fibrosis by changing the level of glycolysis in proximal TECs in a mouse model of UUO (Ref. 99). Lemos *et al*. found that IL-1*β* facilitates Myc-dependent metabolic switch from OXPHOS to glycolysis enzymes in kidney stromal cells, which accelerates the development of tubulointerstitial fibrosis (Ref. 100). It is reported that hypoxia plays a crucial role in the progression of renal fibrosis in ESRD (Refs 101, 102, 103, 104). Hypoxia-responsive signalling pathways including pathways mediated by HIF, TGF-*β* and NF-*κ*B leading to renal fibrosis had been well reviewed (Ref. 105), which also regulate cellular metabolism. For instance, HIF-1*α* is crucial for anaerobic glycolysis of immune cells, increasing glycolysis metabolism in NK cells and enhancing IFN-*γ* production, which in turn aggravates renal fibrosis (Ref. 49) (Fig. 2). TGF-*β* enhances glutamine metabolism, which causes lung fibroblasts by facilitating collagen protein synthesis (Ref. 106). Besides, TGF-*β* is a strong activator of glycolysis in mesenchymal cells, it triggers the cellular metabolism shift towards glycolysis (Refs 107, 108). The pentose phosphate pathway (PPP) is related to an increased requirement for R-5-P and NADPH, utilised in nucleotide synthesis and reductive biosynthesis, respectively. Increased functional activity PPP is associated with diabetic renal hypertrophy (Ref. 109) and renal cell carcinomas (Ref. 110). Oxidative metabolism of glutamine produces citrate and acetyl-CoA for lipid synthesis. A key metabolic hallmark of renal cell carcinoma is increased glutamine utilisation compared to normal kidney tissues (Ref. 111). However, how glutamine metabolism alters in CKD has not been well researched.

In addition to changes in glucose metabolism and glutamine metabolism, it has been confirmed that defective FAO in kidney TECs is tightly associated with renal fibrotic damage (Ref. 112). FAO is a critical energy source for renal TECs and other high-energy demanding cells such as cardiac myocytes (Refs 112, 113). However, excess accumulation of triglycerides induces cellular lipotoxicity. It was uncovered that renal fibrosis is associated with decreased FAO, whereas improving FAO in mouse models of renal fibrosis reduced fibrotic injury (Ref. 112). Fierro-Fernandez *et al*. demonstrated that in the UUO mouse, miR-9-5p protects the kidney by mitigating the downregulation of genes associated with significant metabolic pathways, such as OXPHOS, glycolysis and FAO (Ref. 114). Furthermore, impairment of peroxisome proliferator-activated receptor *α* and the FAO pathway in the renal tubule epithelium aggravates age-associated renal fibrosis (Ref. 115). In diabetic kidney disease, metabolism reprogrammes shift from FAO to glycolysis in TECs and HIF-1*α* plays a key role in this progress, dapagliflozin prevents the high glucose-induced metabolic shift in TECs by inhibiting HIF-1*α* (Ref. 116). The amount of these studies suggested that lipotoxicity via impaired fatty acid oxidisation metabolism could trigger cell death, and inflammation and aggravate CKD (Ref. 117).

## Treatments targeting immune cells metabolic signature in CKD

Targeting immune or non-immune cell metabolism can promote or retard the progression of CKD (Table S1). For instance, tryptophan metabolism exerts immunosuppression effects by depleting tryptophan and increasing immunosuppressive metabolites in the kynurenine pathway (Ref. 118). Overexpressing tryptophan-degrading enzyme IDO in DCs alleviates renal damage in the IgAN mouse model (Ref. 27). Treatment with glycolysis inhibitors (dichloroacetate or shikonin) in M1 macrophages alleviates renal fibrosis in the UUO mouse model (Ref. 44). Furthermore, pharmacological mTOR inhibitors inhibit glycolysis of NK cells both in vivo and in vitro, thus ameliorating mice renal fibrosis in the UUO model (Refs 49, 50). Besides, treatment with mTOR inhibitor rapamycin, which inhibits glycolysis, can reduce the proportion of Th1, Th2 and Th17 cells and boost the proportion of DCs in the kidney to alleviate kidney damage in BALB/C mice with SLE (Ref. 62). However, in addition to inhibiting metabolic pathways of immune cells that can alleviate renal damage or renal fibrosis, so do non-immune cells. Glycolysis inhibitors shikonin and 2-deoxyglucose mitigate renal interstitial fibroblasts activation and renal fibrosis in the UUO mouse model (Ref. 119). Likewise, tsc1-associated mTORC1 signalling activation or treatment with glycolysis inhibitor 2-deoxyglucose attenuates TEC proliferation and kidney fibrosis (Ref. 99). Kang *et al*. found that fenofibrate administration protected mice from the development of renal tubular epithelial fibrosis by strongly inducing transcriptional expression of FAO-related genes in a folate-induced injury model and a UUO model (Ref. 112). Nevertheless, there are already numerous pieces of research in experimental mouse models of kidney diseases but few in clinical studies. In the near future, a large number of clinical studies of inhibitors or activators that directly act on metabolic pathways are called for.

## Summary and outlook

In brief, immunometabolism as an emerging hot spot has a significant role in cancer as well as acute or chronic immune disease. It is significant and urgent to make clear the metabolic signature correlations between immune cells and parenchymal cells in the kidney. Yet, the intricate connection between immune or non-immune cells and their metabolic features has not been fully studied. In this review, we describe the metabolic reprogramming of immune cells and parenchymal cells during pathogenic processes in immune-related kidney diseases. The agents that directly act on some metabolites, metabolic pathway regulators or rate-limiting enzymes may become a novel direction for the treatment of CKD.

## Supplementary material

For supplementary material accompanying this paper visit http://doi.org/10.1017/erm.2022.35. click here to view supplementary material
