# Supplementary material for: Metabolic signatures of immune cells in chronic kidney disease
Source: Expert Rev Mol Med. 2022 Oct 21;24:e40. doi: 10.1017/erm.2022.35 (PMC9884772; doi:10.1017/erm.2022.35)
Supplement: Supplementary file 1 [file S1462399422000357sup001.docx]

**Table S1.** Influences of kidney disease progression by interfering with immune and non-immune cell metabolic pathways in literature

| **Human/Mouse** | **Disease model** | **Cells** | **Treatment** | **Disrupted metabolic pathways** | **Renal Outcomes** | **Ref.** |
| --- | --- | --- | --- | --- | --- | --- |
| Mouse | IgAN | DCs | Overexpression IDO | Tryptophan  metabolic | Alleviate injury | (27) |
| Mouse | UUO | Macrophages | Dichloroacetate/  shikonin | Glycolysis | Alleviate fibrosis | (44) |
| Mouse | UUO | NK cells | mTOR inhibitors | Glycolysis | Alleviate fibrosis | (49,50) |
| BALB/C mice | SLE | Th1/Th2/Th17/DCs | rapamycin | Glycolysis | Alleviate injury | (63) |
| Mouse | UUO | Fibroblasts | shikonin and  2-deoxyglucose | Glycolysis | Alleviate fibrosis | (119) |
| Mouse | UUO | proximal TECs | Tsc1 | Glycolysis | Alleviate fibrosis | (99) |
| Mouse | Folate-induced injury model/UUO | TECs | fenofibrate | FAO | Alleviate fibrosis |  |

IgAN, IgA nephropathy; DCs, dendritic cells; IDO, indoleamine 2,3-dioxygenase; l, Tsc1, tuberous sclerosis complex 1; TECs, tubular epithelial cells; FAO, fatty acid oxidation.
